# Supplementary material for: In the Murine and Bovine Maternal Mammary Gland Signal Transducer and Activator of Transcription 3 is Activated in Clusters of Epithelial Cells around the Day of Birth
Source: J Mammary Gland Biol Neoplasia. 2024 May 9;29(1):10. doi: 10.1007/s10911-024-09561-5 (PMC11081984; doi:10.1007/s10911-024-09561-5)
Supplement: Supplementary file 1 — Supplementary Material 1 [file 10911_2024_9561_MOESM1_ESM.docx]

**Online Resources:**

**In the murine and bovine maternal mammary gland signal transducer and activator of transcription 3 is activated in clusters of epithelial cells around the day of birth**

**Online resource 1. Around the day of birth there is polarisation of alveoli towards either a low- or high- proportion of pSTAT3 positive alveolar epithelial cells.** (a, b) Murine tissue from 17.5 dG (a) and 2 dL (b). IHC for pSTAT3 (brown) with haematoxylin counterstain. Arrows indicate rare foci of epithelial pSTAT3 positivity. Scale bar = 400 μm.

**
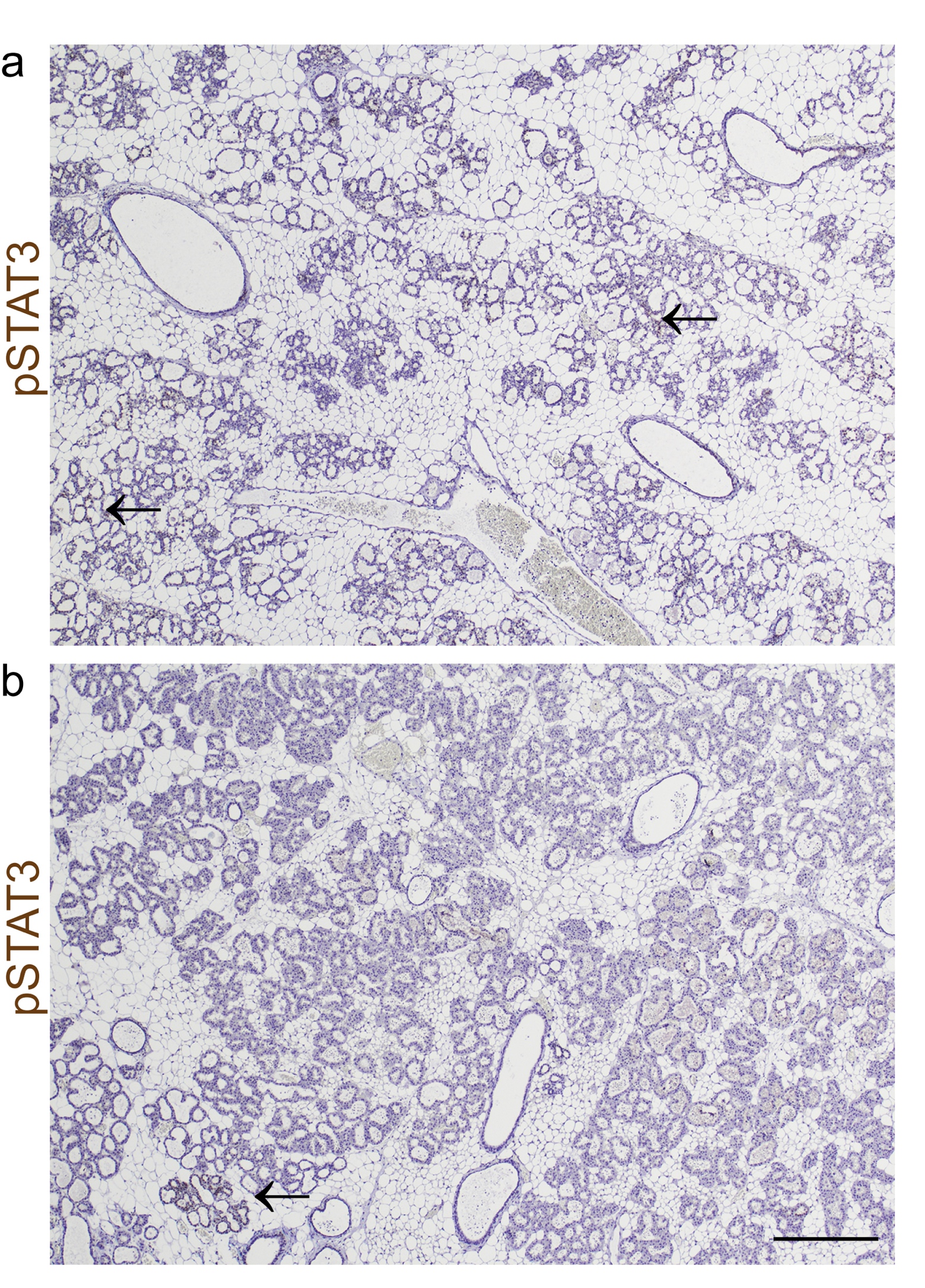
**

**Online resource 2. Table detailing bovine samples used in this study.**

dG days gestation; dL days lactation; HF Holstein Friesian.

| Case number | Age (years) | Breed | Mammary postnatal developmental stage | Cause of death (d) or euthanasia (e) |
| --- | --- | --- | --- | --- |
| 1 | 2 | HF | 248 dG | Ketosis and hepatic lipidosis (d). |
| 2 | 2.25 | HF | 250 dG | Ketosis, hepatic lipidosis and inter-mandibular cellulitis (e). |
| 3 | 2.5 | Belgian Blue cross | 1 dL | Vaginal haemorrhage and tear (d). |
| 4 | 2 | Aberdeen Angus | 1 dL | Broken leg (e). |
| 5 | >4 | HF | 2-3 dL | Chronic milk fever and jejunal infarction (d). Chronic mastitis noted (left fore). |
| 6 | 7 | HF | 8 dL | Ruptured meniscus hindlimb (e). |
| 7 | 4 | HF | 46 dL | Haemorrhagic enteritis (d).  Chronic mastitis noted (left fore). |

**Online resource 3. The mammary gland of cows in the last third of gestation, and in early lactation, exhibits variable levels of mammary alveolar development and pSTAT3 expression.** Bovine tissue from 8 dL (case 7). (a) Alveoli are multifocally expanded with proteinaceous secretion (arrows). (b) pSTAT3 is multifocally expressed in ducts (arrow). (c) Multifocally there is intense pSTAT3 expression (c) but in other locally extensive foci there is minimal pSTAT3 expression (d). Haematoxylin and eosin staining (a) and IHC for pSTAT3 (brown) with haematoxylin counterstain (b, c, d). Scale bar = 400 μm (a) and 80 μm (b, c, d).


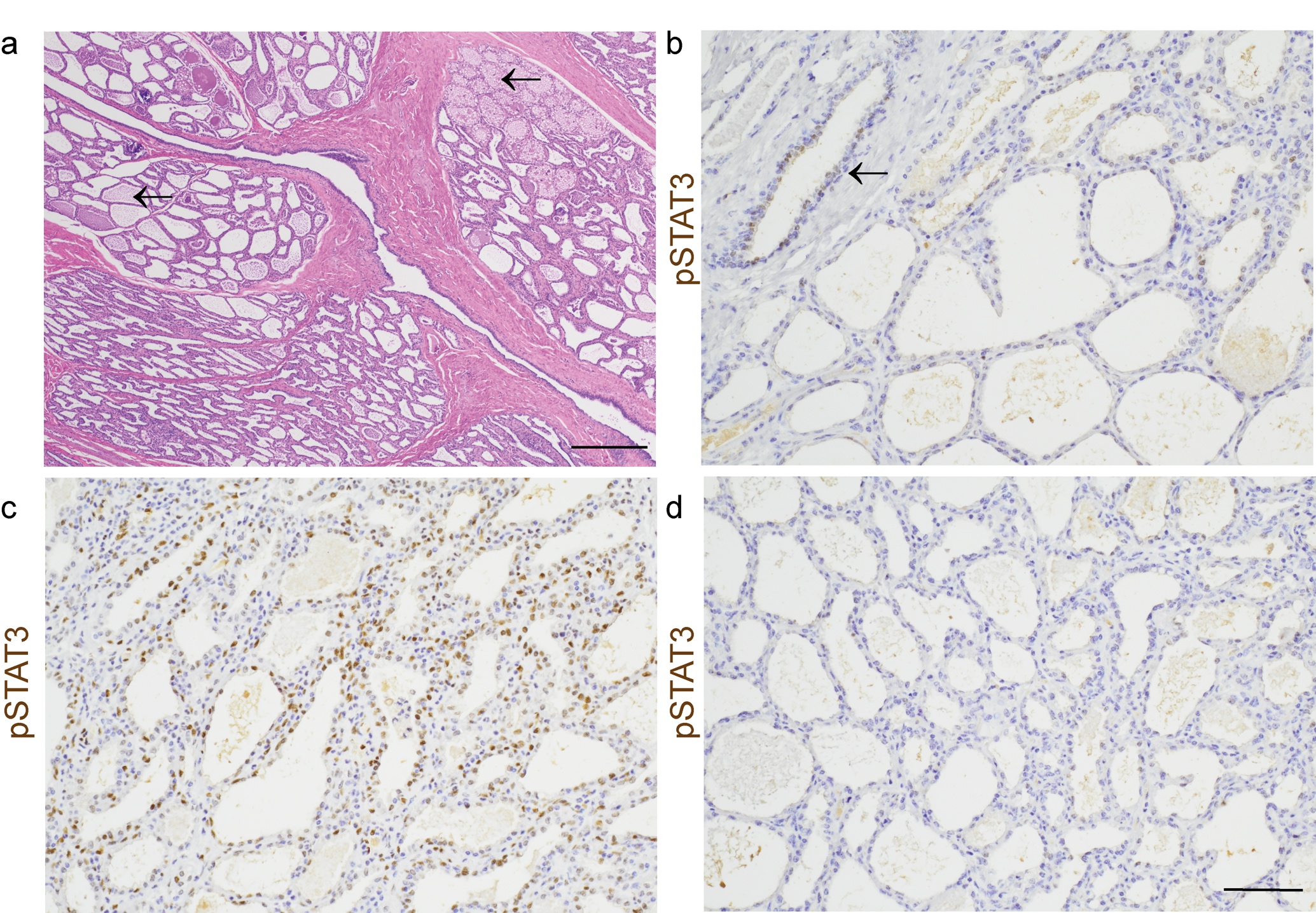


**Online resource 4.** **Expression of pSTAT3 is unaffected by mammary alveolar dimensions.** Scatter plots demonstrating that the distribution of alveolar lengths (a) and widths (b) does not differ significantly between alveoli without any luminal epithelial pSTAT3 expression (denoted negative) and those with any degree of luminal epithelial pSTAT3 expression (denoted positive). Dots represent mean alveolar dimensions per quarter for 12 quarters from 7 cows (negative alveoli) and 7 quarters from 4 cows (positive alveoli). Bars represent mean ± standard deviation; ns, not significant.

**
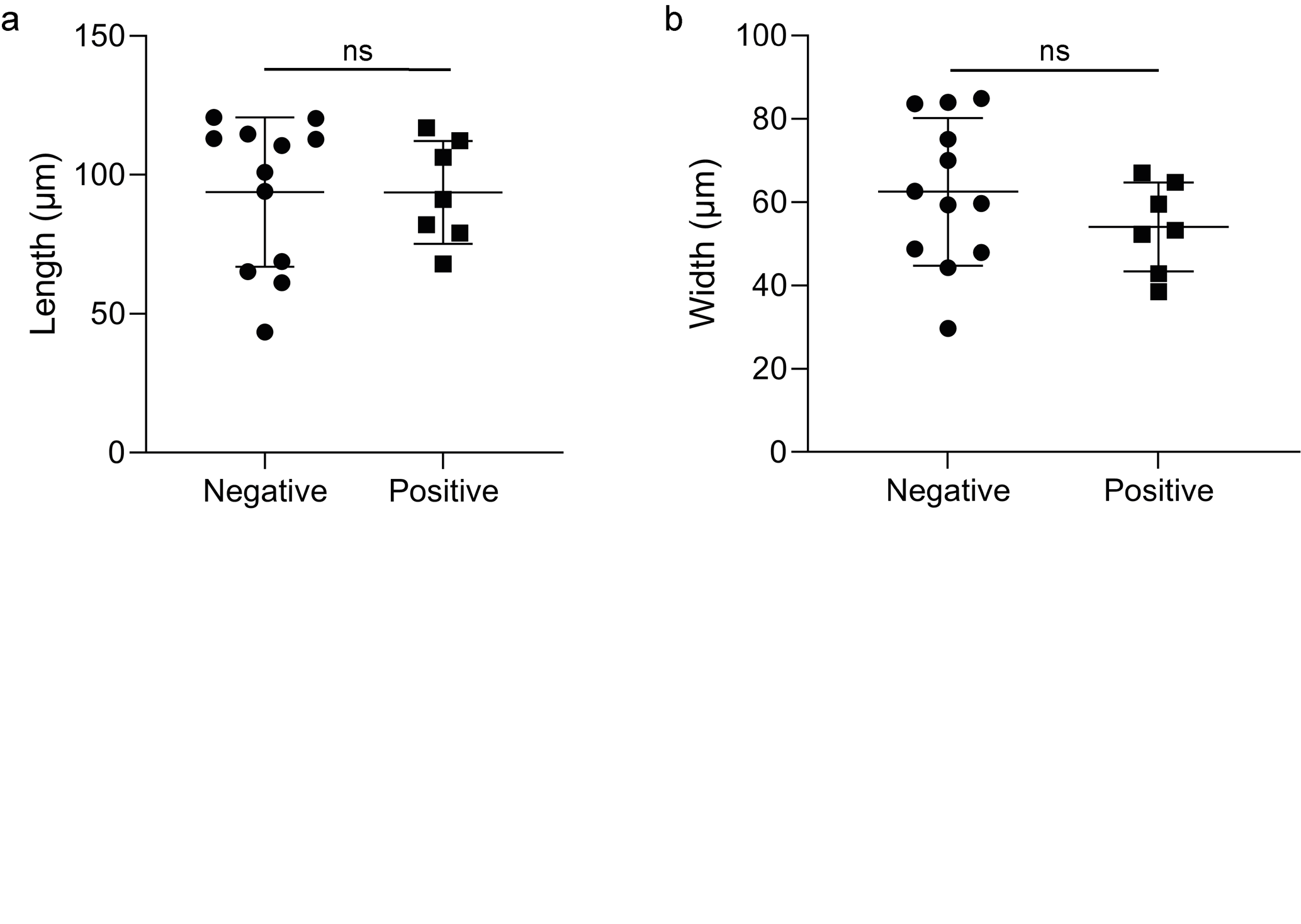
**
